# Uncertainty Analysis in the Calibration of an Emission Tomography System for Quantitative Imaging

**DOI:** 10.1155/2017/9830386

**Published:** 2017-10-12

**Authors:** Marco D'Arienzo, Maurice Cox

**Affiliations:** ^1^ENEA, National Institute of Ionizing Radiation Metrology, Via Anguillarese 301, 00123 Rome, Italy; ^2^National Physical Laboratory, Hampton Road, Teddington TW11 0LW, UK

## Abstract

It is generally acknowledged that calibration of the imaging system (be it a SPECT or a PET scanner) is one of the critical components associated with in vivo activity quantification in nuclear medicine. The system calibration is generally performed through the acquisition of a source with a known amount of radioactivity. The decay-corrected calibration factor is the “output” quantity in a measurement model for the process. This quantity is a function of a number of “input” variables, including total counts in the volume of interest (VOI), radionuclide activity concentration, source volume, acquisition duration, radionuclide half-life, and calibration time of the radionuclide. Uncertainties in the input variables propagate through the calculation to the “combined” uncertainty in the output quantity. In the present study, using the general formula given in the GUM (Guide to the Expression of Uncertainty in Measurement) for aggregating uncertainty components, we derive a practical relation to assess the combined standard uncertainty for the calibration factor of an emission tomography system. At a time of increasing need for accuracy in quantification studies, the proposed approach has the potential to be easily implemented in clinical practice.

## 1. Introduction

Positron emission tomography (PET) and single-photon emission computed tomography (SPECT) provide a means of evaluating the biological function of cells and organs, producing three-dimensional images of the distribution of radiopharmaceuticals introduced into the patient's body. These molecular imaging techniques rely on radiolabelled molecules (generally consisting of a radionuclide and a molecule that determines the localization) that build up in areas of disease allowing for the collection of metabolic and functional information in vivo.

Over the last decade radionuclide imaging has gained popularity as a quantitative technique. In particular, recent advances in image processing software and the advent of hybrid SPECT/CT and PET/CT scanners have paved the way for accurate quantitative analysis, that is, the determination of activity concentration within a given tissue of interest in absolute units, for example, becquerel per millilitre or becquerel per cubic centimetre.

PET was developed as a quantitative tool and the standardized uptake value (SUV) is probably the most widely used indicator for the quantification of ^18^F-FDG PET studies. SUV is a measure of how much cellular activity occurs in the region of uptake and is mathematically defined as the concentration of the radionuclide in the volume of interest (VOI) divided by the injected activity normalized for the patient's body weight. High SUV values are likely to represent pathological conditions, from inflammation to infection to cancer, with higher numbers being most suggestive of cancer.

On the other hand, SPECT has traditionally been seen as nonquantitative. This is because quantification using SPECT images is a time-consuming process, requiring accurate methods that correct for a number of degrading factors, among which are attenuation, scatter, dead time, and partial volume effects. However, the advent of hybrid SPECT/CT scanners has made quantitative SPECT viable in a manner similar to quantitative PET. An extensive review of potential uses for quantitative SPECT is given in [1].

Calibration of the imaging system is an essential prerequisite to convert reconstructed voxel values to absolute activity or activity concentration, both in SPECT and in PET. As a consequence, it is generally acknowledged that calibration of the imaging system in emission tomography is a critical requirement for producing accurate quantitative data both in diagnosis and in therapy [2].

Gamma camera calibration can be performed either in air or in water. Calibration in air consists of determining the gamma camera sensitivity through the acquisition of a small volume of activity, for example, point-like source, Petri dish, line source, or spherical source. On the other hand, gamma camera calibration in water involves the use of an extended volume source mimicking the clinical conditions encountered in patient studies [3]. The general formalism for the evaluation of the system calibration factor is given in NEMA publication NU 1-2012 [4]. Absolute calibration of the PET system (often referred to as “well counter calibration”) is generally performed by scanning a large water-filled phantom that contains a known amount of activity. This procedure allows counts per second to be transformed to activity concentration. Following the absolute activity calibration, the voxel intensity in any PET image is divided by the calibration factor to obtain calibrated images in terms of Bqcm^−3^. Further details on the procedure for evaluating the performance of positron emission tomographs are reported in NEMA Standards Publication NU 2-2007 [5].

Without loss of generality, the decay-corrected scanner calibration factor *S*_rc_ in emission tomography, the output quantity, can be written in terms of the input quantities

 
*R*, the summed counts over a given VOI in the image
 
*V*, the volume of interest
 
*C*_*a*_, the radionuclide activity concentration
 
*T*_0_, the acquisition start time
 
*T*_cal_, the time of activity calibration
 
*T*_1/2_, the radionuclide physical half-life
 
*T*_acq_, the acquisition duration

as follows [4, 5]:

(1) Src=RVCaexp⁡T0−TcalT1/2ln⁡2ln⁡2T1/2·1−exp−TacqT1/2ln⁡2−1.

In this formalism all input quantities have associated standard uncertainties, which propagate through the calculation to the “combined” standard uncertainty *u*(*S*_rc_) in the output quantity. In the present study, using the general approach proposed in the GUM (Guide to the Expression of Uncertainty in Measurement) [7] for combining uncertainty components (the “law of propagation of uncertainty”), we derive a relation to assess the combined standard uncertainty for the calibration factor.

## 2. Evaluation of Uncertainty

Let *Q*_1_, *Q*_2_,…, *Q*_*n*_ denote a set of *n* “input” quantities and *Y* an “output” quantity or measurand. The GUM [7] considers the generic measurement model

(2) $$\begin{array}{c} Y=f\left(\;Q_{1},Q_{2},\ldots ,Q_{n}\;\right), \end{array}$$

that is, a known functional relationship between the input and the output quantities. Given estimates *q*_1_, *q*_2_,…, *q*_*n*_ of the input quantities, the GUM uses

(3) $$\begin{array}{c} y=f\left(\;q_{1},q_{2},\ldots ,q_{n}\;\right) \end{array}$$

as the corresponding estimate of *Y*. Further, given standard uncertainties *u*(*q*_1_), *u*(*q*_2_),…, *u*(*q*_*n*_) associated with *q*_1_, *q*_2_,…, *q*_*n*_, the GUM applies the law of propagation of uncertainty (LPU) to evaluate the combined standard uncertainty *u*(*y*) associated with *y*. For independent input quantities, LPU is described by the following expression:

(4) u2y=∂f∂q12u2q1+∂f∂q22u2q2+⋯+∂f∂qn2u2qn,

in which ∂*f*/∂*q*_*i*_ denotes ∂*f*/∂*Q*_*i*_ evaluated at *q*_1_, *q*_2_,…, *q*_*n*_.

Therefore, with reference to expression (1), an estimate of the calibration factor *S*_rc_ is given by evaluating this expression for estimates of the input quantities *R*, *V*, *C*_*a*_, *T*_0_, *T*_cal_, *T*_1/2_, and *T*_acq_, which are assumed independent. Further, the standard uncertainty *u*(*S*_rc_) associated with this estimate of *S*_rc_ is given by the following (for notational simplicity we do not distinguish between a quantity and an estimate of the quantity):

(5) u2Src=∂Src∂R2u2R+∂Src∂V2u2V+∂Src∂Ca2u2Ca+∂Src∂T02u2T0+∂Src∂Tcal2u2Tcal+∂Src∂T1/22u2T1/2+∂Src∂Tacqu2Tacq.

Although the use of expression (5), after evaluating the necessary partial derivatives, will deliver the required standard uncertainty *u*(*S*_rc_), this uncertainty can be obtained more conveniently by rewriting expression (1). Using the substitutions,

(6) X1=RVCa,

(7) X2=T0−Tcal,

(8) X3=ln⁡2T1/2,

(9) X4=Tacq,

(1) can be expressed as

(10) $$\begin{array}{c} S_{rc}=X_{1}\exp\left(\;X_{2}X_{3}\;\right)X_{3}{\left[\;1-exp\left(\;-X_{3}X_{4}\;\right)\;\right]}^{-1}. \end{array}$$

Since each quantity on the right sides of equations (6) to (9) does not appear in any others of these equations, the independence of these quantities implies the independence of *X*_1_, *X*_2_, *X*_3_, and *X*_4_. Hence, in place of expression (5) we have

(11) u2Src=∂Src∂X12u2X1+∂Src∂X22u2X2+∂Src∂X32u2X3+∂Src∂X42u2X4.

Defining

(12) α=T0−TcalT1/2ln⁡2=X2X3,

(13) β=TacqT1/2ln⁡2=X3X4,

each partial derivative appearing in expression (11) can simply be expressed in terms of *S*_rc_, *X*_1_, *X*_2_, *X*_3_, *X*_4_, *α*, and *β*:

(14) ∂Src∂X1=Src1X1,∂Src∂X2=SrcαX2,∂Src∂X3=Src1+α1−e−β−βe−βX31−e−β,∂Src∂X4=−Srcβe−βX41−e−β.

Expression (11) can then be recast as

(15) u2SrcSrc2=uX1X12+α2uX2X22+1+α1−e−β−βe−β1−e−β2uX3X32+βe−β1−e−β2uX4X42,

or, in terms of relative standard uncertainties, where, for instance, *u*_rel_(*X*_1_) denotes *u*(*X*_1_)/|*X*_1_|  (*X*_1_ ≠ 0),

(16) urel2Src=urel2X1+α2urel2X2+1+α1−e−β−βe−β1−e−β2urel2X3+βe−β1−e−β2urel2X4.

It is noted that for most radionuclides the acquisition time is generally much smaller than the radionuclide half-life (i.e., *T*_acq_ ≪ *T*_1/2_ and, with reference to expression (13), *β* ≪ 1). This is especially true for therapeutic radionuclides, whose half-life is typically a few days, while acquisition times are generally in the range of 10 min to 30 min. Recalling that *e*^−*β*^ ≈ 1 − *β* provided *β* is reasonably small, (16) can be written in simplified form:

(17) urel2Src≈urel2X1+α2urel2X2+α2urel2X3+urel2X4.

Computing the combined standard uncertainty on the calibration factor according to (17) requires minimal effort and has the potential to be easily implemented in clinical practice. The following paragraphs describe how each source of uncertainty appearing in expression (17) can be practically and effectively estimated.

### 2.1. Evaluation of *u*_rel_(*X*_1_)

Using a variant of the law of propagation of uncertainty that applies to a model in product/quotient form [7], the squared relative standard uncertainty on the term *X*_1_ (see (6)) can be expressed as the sum of the squares of the relative standard uncertainties in each of the quantities appearing in (6):

(18) $$\begin{array}{c} u_{rel}^{2}\left(\;X_{1}\;\right)=u_{rel}^{2}\left(\;R\;\right)+u_{rel}^{2}\left(\;V\;\right)+u_{rel}^{2}\left(\;C_{a}\;\right). \end{array}$$

In turn the radionuclide activity concentration *C*_*a*_ is given by the absolute activity *A* of the radionuclide divided by the volume *V*_liq_ of the liquid solution in which it is dispersed; namely,

(19) $$\begin{array}{c} C_{a}=\frac{A}{V_{liq}}. \end{array}$$

Thus, (18) can be rewritten as

(20) $$\begin{array}{c} u_{rel}^{2}\left(\;X_{1}\;\right)=u_{rel}^{2}\left(\;R\;\right)+u_{rel}^{2}\left(\;V\;\right)+u_{rel}^{2}\left(\;A\;\right)+u_{rel}^{2}\left(\;V_{liq}\;\right). \end{array}$$

Considerations on how the above uncertainties can be evaluated are given below.

(i) The relative standard uncertainty *u*_rel_(*R*) of the counts *R* in a given VOI depends on both physical factors affecting activity quantification and the noise level generated during the process of reconstructing the image. Physical factors include photon interactions in the patient, loss of spatial accuracy due to limited system resolution, partial volume effects, and noise resulting from the random nature of radioactive decay and absorption. Counting statistics and acquisition time play an additional role. Both in SPECT [8] and in PET [9] the coefficient of variation, that is, the ratio of the standard deviation to the average signal measured in the VOI, is a viable approach to assessing the noise level.
(ii) The relative standard uncertainty *u*_rel_(*V*) is generated by the voxelization of the VOI, that is, the process of converting the continuous geometric representation of the VOI into a set of voxels that approximates the continuous object. By simply selecting all voxels that are intersected by the continuous VOI (that may, e.g., be approximately spherical or cylindrical), the generated digital object may as a result be too coarse, including more or fewer voxels than are necessary.
For simplicity, we consider a spherical VOI represented in terms of cubical voxels of side *ℓ*. We consider an indicative standard uncertainty of the diameter *d* to be size of a voxel; namely, *u*(*d*) = *ℓ*. It follows that the standard uncertainty *u*(*r*) associated with the radius *r* of the sphere will be half the side of the voxel; that is, *u*(*r*) = *u*(*d*)/2 = *ℓ*/2. The standard uncertainty *u*(*r*) translates into a standard uncertainty *u*(*V*) associated with the volume delineated by the VOI. For some constant *C*, *V* = *Cr*^3^. Hence d*V*/d*r* = 3*Cr*^2^, and, accordingly,
  (21) $$\begin{array}{c} u_{rel}\left(\;V\;\right)=\frac{u\left(V\right)}{V}=3\frac{u\left(r\right)}{r}=3u_{rel}\left(\;r\;\right). \end{array}$$
Thus, a relative standard uncertainty associated with a radius *r* induces a relative standard uncertainty associated with a volume *V* that is three times as large. For instance, for a plan view of a sphere of some 150 mm in radius with a voxel side equal to 3 mm, *u*(*r*) = 1.5 mm. Thus, *u*_rel_(*r*) ≈ 1% and *u*_rel_(*V*) ≈ 3%.
In practice the VOI will differ from a sphere, but a mean radius *r* of the VOI can instead be considered. The standard uncertainty *u*(*r*) translates into a standard uncertainty *u*(*V*) associated with the volume *V* delineated by the VOI, and the relationship *V* = *Cr*^3^ still holds.
(iii) *u*_rel_(*A*) is the relative standard uncertainty associated with activity measurement. At a clinical level, activity measurements are generally made using commercially available radionuclide calibrators traceable to a national standards laboratory for the geometry being measured. The typical instrument for assaying radiopharmaceuticals is the pressurized, well-type ionization chamber. These instruments are capable of providing radioactivity measurements with varying degrees of accuracy, depending on the radionuclide and the sample configurations (e.g., glass vials and/or plastic syringes).
From a regulatory standpoint, in most countries the standard of good practice is that the administered activity should be within 10% of the prescribed activity [10]. As a consequence, given the other sources of error involved in the administration of the radiopharmaceutical, radionuclide calibrators should provide an expanded uncertainty below 10%, perhaps 5% (for a coverage factor of *k* = 2, giving approximately 95% coverage). The achievable uncertainty in clinical practice is reported in the AAPM guidelines [11]. For radionuclide calibrator field instruments an expanded uncertainty no greater than 5% is recommended for photon emitters > 100 keV. An expanded uncertainty no greater than 10%  (*k* = 2) for photon emitters < 100 keV is recommended [11]. For medium and high-energy beta emitters, a radionuclide calibrator expanded uncertainty no greater than 5%  (*k* = 2) is suggested, while for low-energy beta emitters an expanded uncertainty no greater than 10%  (*k* = 2) is recommended [11]. Secondary standard radionuclide calibrators and reference radionuclide calibrators should be calibrated to be within an expanded uncertainty no greater than 2%  (*k* = 2) for photon emitters > 100 keV and medium and high-energy beta emitters. For the same instruments an expanded uncertainty no greater than 5%  (*k* = 2) is recommended for photon emitters < 100 keV and low-energy beta emitters [11].
(iv) The relative standard uncertainty *u*_rel_(*V*_liq_) is associated with volume measurement, which typically translates into weighing of masses. As a general rule, the significant factors that contribute to measurement uncertainty across the weighing range are repeatability, eccentricity (the error associated with not placing the weight in the centre of the weighing pan), nonlinearity (the error due to the nonlinear behaviour of the balance upon increasing the load on the weighing pan), and sensitivity (i.e., systematic deviation). If analytic balances are used for the measurements of small masses, uncertainties below 0.001% can be achieved [12]. If large masses need to be weighed (e.g., large phantoms filled with water mixed with radionuclide) laboratory balances with weighing capacity of up to 100 kg to 150 kg are commercially available, yielding typical relative standard uncertainties below 0.05%.

### 2.2. Evaluation of *u*_rel_(*X*_2_)

The relative standard uncertainty *u*_rel_(*X*_2_) is associated with a possible time offset between the clocks used to assess the reference calibration time *T*_cal_ and the acquisition start time *T*_0_. With reference to (7),

(22) $$\begin{array}{c} u_{rel}\left(\;X_{2}\;\right)=\frac{u\left(T_{0}-T_{cal}\right)}{T_{0}-T_{cal}}, \end{array}$$

where *u*(*T*_0_ − *T*_cal_) is the standard uncertainty associated with the time difference between the acquisition start time *T*_0_ and the reference calibration time *T*_cal_. The absolute time offset between the two clocks used to determine *T*_0_ and *T*_cal_ can be considered representative of *u*(*T*_0_ − *T*_cal_).

It is worth noting that the overall impact of the time offset on the calibration factor uncertainty does not depend on the absolute time difference *T*_0_ − *T*_cal_. In fact, with reference to (16) (also see (12)), the absolute time difference cancels leaving a dependence solely on the terms *u*(*T*_0_ − *T*_cal_) and *T*_1/2_:

(23) α2urel2X2=T0−TcalT1/2ln⁡22uT0−TcalT0−Tcal2=uT0−TcalT1/2ln⁡22.

**Figure 1:**
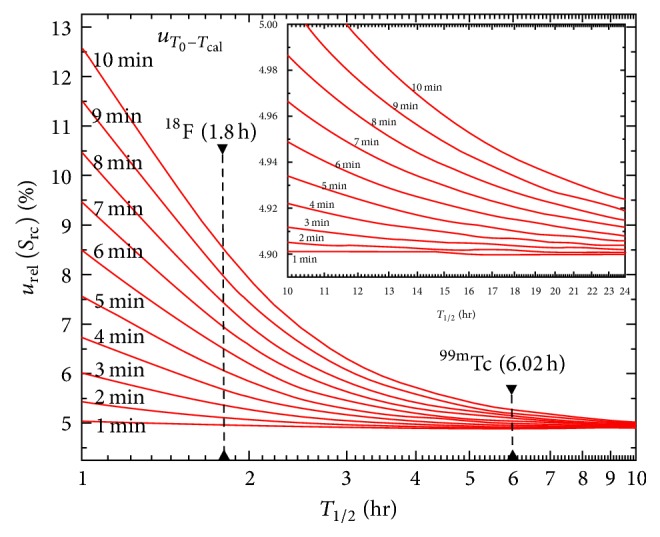
Impact of time offset on the final relative standard uncertainty, *u*_rel_(*S*_rc_), as a function of radionuclide half-life *T*_1/2_. The graph shows the calibration factor relative standard uncertainty for different values of *u*(*T*_0_ − *T*_cal_). The case for short-lived radionuclides is shown.

**Figure 2:**
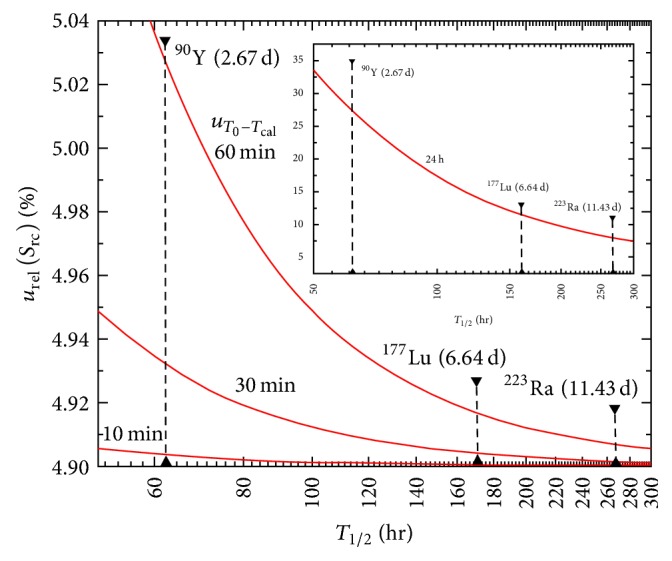
Impact of time offset on the final relative standard uncertainty, *u*_rel_(*S*_rc_), as a function of radionuclide half-life *T*_1/2_. The graph shows the calibration factor relative standard uncertainty for different values of *u*(*T*_0_ − *T*_cal_). The case for long-lived radionuclides is shown.

Figure 1 illustrates the impact of time offset on the final relative standard uncertainty, *u*_rel_(*S*_rc_), as a function of radionuclide half-life for short-lived radionuclides. The case for two widely used diagnostic radionuclides, ^18^F (*T*_1/2_ = 1.8 h) and ^99m^Tc (*T*_1/2_ = 6.02 h), is shown. *u*_rel_(*S*_rc_) as obtained from (16) is plotted as a function of the radionuclide half-life for different values of *u*(*T*_0_ − *T*_cal_), and the absolute time offset between the two clocks is used to determine *T*_0_ and *T*_cal_. With reference to (16) and (18), the following relative standard uncertainties were considered: *u*_rel_(*R*) = 4%, *u*_rel_(*V*) = 2%, *u*_rel_(*A*) = 2%, *u*_rel_(*X*_3_) = 0.05%, and *u*_rel_(*X*_4_) = 0.1%. As a general rule, the greater the ratio between the time offset and the radionuclide half-life is, the larger the impact on the calibration factor relative standard uncertainty is.  Figure 2 reports the same data for long-lived radionuclides, for example, ^90^Y (*T*_1/2_ = 64 h), ^177^Lu (*T*_1/2_ = 160 h), and ^223^Ra (*T*_1/2_ = 274 h). The extreme case of *u*(*T*_0_ − *T*_cal_) = 24 h is presented.

### 2.3. Evaluation of *u*_rel_(*X*_3_)

With reference to (8), *u*_rel_(*X*_3_) represents the relative standard uncertainty of the radionuclide half-life *T*_1/2_:

(24) $$\begin{array}{c} u_{rel}\left(\;X_{3}\;\right)=u_{rel}\left(\;T_{1/2}\;\right). \end{array}$$

The calibrated activity value for a standard (as for any instrument) refers to a fixed reference date, and the half-life must be known with sufficient accuracy in order to calculate the activity at the time the standard (or instrument) is used, which may well be several half-lives later. Metrology of radionuclide activity is a mature science and many data are currently available with sufficiently small uncertainties for most practical purposes.  Table 1 lists adopted half-life values and associated uncertainties for a selection of radionuclides used both in diagnosis and in therapy. The relative standard uncertainty of the half-life of radionuclides typically used in clinical practice is well below 0.1%.

### 2.4. Evaluation of *u*_rel_(*X*_4_)

With reference to (9), *u*_rel_(*X*_4_) is the relative standard uncertainty associated with the overall acquisition time *T*_acq_:

(25) $$\begin{array}{c} u_{rel}\left(\;X_{4}\;\right)=u_{rel}\left(\;T_{acq}\;\right). \end{array}$$

Both in SPECT and in PET, the acquisition time is defined by the user and it is computer-controlled by the workstation software. Therefore, assuming that the software accesses the clock correctly, the accuracy of timing functions is determined by the accuracy of the clock itself. Therefore, timing tests are recommended within the Quality Assurance programme, consisting in the verification of the acquisition time recorded by the computer [13]. In clinical practice accurate estimates of the scan time can generally be achieved and this uncertainty component is likely to make negligible contribution to the uncertainty in the calibration factor. Caution is advised in the presence of dynamic studies in SPECT, where timing errors between frames may become considerable at high counting and framing rate [13].

### 2.5. Calibration Factor Relative Standard Uncertainty

Equation (17) gives a simplified form for the relative standard uncertainty *u*_rel_(*S*_rc_) in the calibration factor *S*_rc_. By combining this equation and (20), (22), (24), and (25) the combined standard uncertainty in the calibration factor of an emission tomography system can be obtained. In terms of relative standard uncertainties,

(26) urel2Src≈urel2R+urel2V+urel2A+urel2Vliq+T0−Tcalln⁡2T1/22·urel2T0−Tcal+urel2T1/2+urel2Tacq.

Equation (26) has the potential to be easily implemented in clinical practice to assess the combined relative standard uncertainty *u*_rel_(*S*_rc_) in the calibration factor of an emission tomography system.

### 2.6. Additional Sources of Uncertainty Affecting Quantification in Emerging PET Imaging Modalities

There are a number of factors affecting the accuracy of image-based activity estimates, both in SPECT and in PET. Attenuation and scatter of photons degrade the image quality and the accuracy of activity estimates varies with the object size due to the limited spatial resolution, dead time, and partial volume effects. The presence of random coincidences plays an additional role in PET and it is currently the subject of ongoing research in PET imaging. As a consequence, proper compensation techniques are required to perform accurate absolute quantification with emission tomography. A detailed description of all possible sources of uncertainty in activity quantification falls outside the scope of this paper and the reader is referred to [14] for an extensive review on this topic.

Attenuation is the loss of events because of their absorption in the body and it is by far one of the main factors impacting quantification studies. In hybrid SPECT/CT systems CT data can be used to correct for attenuation on a slice-by-slice basis. Of note, because attenuation varies with photon energy, it is necessary to rescale the CT attenuation data to match the energy of the radionuclide used in SPECT. This rescaling is generally accomplished by using a bilinear model relating attenuation coefficients at the desired energy to CT numbers measured at the effective energy of the CT beam of X-rays [15, 16]. Similarly, on PET/CT scanners, the attenuation map is assessed by rescaling CT Hounsfield units to 511 KeV attenuation coefficients. As a consequence, in both hybrid SPECT/CT and PET/CT systems, further uncertainty is likely to be introduced in the quantification process.

Currently, PET coupled with MRI as a hybrid imaging modality is receiving increasing attention and it is likely to become the technology of choice in the future. A different attenuation correction strategy is required in combined PET/MR systems. In fact, the small bore inside the scanner and the strong magnetic field do not permit a rotating CT device to be integrated. As a consequence tissue attenuation information needs to be determined from the MR image [17]. Attenuation correction through MR images is inherently challenging as MRI image intensity correlates with proton density and relaxation properties of tissue, not with tissue density. As an example, bone and cavities present similar signal intensities in MRI. However their density produces the highest and lowest attenuation in PET, respectively. Many studies have been published on correction strategies using MR imaging, including intensity-based tissue type segmentation and classification of an MR image [18–20] and atlas-based segmentation techniques [21, 22]. Attenuation correction through MR images has the potential to introduce quantification inaccuracies. Evaluation of the quantitative accuracy of MR-based attenuation correction in terms of SUV error has been discussed by several authors [23–25]. Of note, resent research has proven that TOF PET has the potential to remarkably reduce attenuation correction artifacts and quantification errors in the lungs and bone tissues [26].

Yet another question of interest is the possible uncertainty introduced by radiation yield and decay branching ratios of radionuclides used in the clinical practice. Against this backdrop, the ^90^Y positron branching ratio deserves special attention. In fact, ^90^Y is traditionally thought of as a pure electron emitter originating from ^90^Y conversion into ^90^Zr (beta decay). However previous studies showed that the decay of ^90^Y has a minor branch to the 0^+^ first excited state of ^90^Zr at 1.76 MeV, that is followed by a *β*^+^/*β*^−^ emission [27]. In recent years, a number of authors have used the small positronic emission of ^90^Y, (3.186 ± 0.047) · 10^−5^ [28], to obtain high-resolution positron emission tomography (PET) images of ^90^Y biodistribution after liver radioembolization. Since the accuracy on the evaluation of the absorbed dose to the patient depends on the knowledge of the ^90^Y positron branching ratio, the accuracy of ^90^Y PET/CT imaging and dosimetry is strongly related to the measurement precision of the internal pair production branching ratio. In the near future, new experimental measurements of the internal pair production branching ratio are desirable in order to achieve more accurate quantification in postradioembolization imaging using PET/CT. A recent joint research project (EMPIR-MRTDosimetry [29]) is currently dealing with this issue. The project will carry out new measurements to determine branching ratios and emission probabilities for  ^90^Y with greater accuracy. This will be the first step for providing greater reliability of  ^90^Y and will enable improved quantitative imaging accuracy and dose estimation for such radiopharmaceutical.

## 3. Discussion

Radionuclide imaging and its quantitative characteristics are increasingly being recognized as providing an objective tool for diagnosis, staging, and therapy response evaluation. In addition, the advent of hybrid SPECT/CT and PET/CT systems and the introduction of relatively new radiopharmaceuticals has generated much interest in activity quantification.

Calibration is the process of establishing the relationship between the measured count rate per volume and activity concentration. Understanding the factors that contribute to the uncertainty in the calibration factor is essential to obtain reliable quantitative information in clinical practice. Interpretation of quantitative data without appreciating the magnitude of the associated uncertainty may result in clinical errors or misleading information.

Over the last few years a number of studies [30, 31] and European projects [29, 32] have been dedicated to the assessment of factors affecting the calibration procedures, both in SPECT and in PET. Against this backdrop, reducing the uncertainty associated with activity quantification has become a key objective in clinical practice, especially in the light of recent technological advances allowing for the possibility of using quantitative data for dosimetry purposes.

In the present study the decay-corrected calibration factor *S*_rc_ was described as a function of a number of “input” variables, whose uncertainties propagate through the calculation to the “combined” uncertainty in the output quantity. Combining (17) and (20) we derived a practical formula, (26), that can be used in clinical practice to assess the combined relative standard uncertainty *u*_rel_(*S*_rc_) in the calibration factor of an emission tomography system.

Our analysis showed that a number of factors may potentially contribute to *u*_rel_(*S*_rc_). In particular, the relative standard uncertainty *u*_rel_(*R*) of the counts *R* in a given VOI and the relative standard uncertainty associated with activity measurements, *u*_rel_(*A*), may play a major role. The latter may lie between 2% and 5% depending on the activity calibrator used. However, if activity is determined by a national metrology laboratory then this uncertainty can be reduced dramatically. Within this context, experimental verification of the accuracy of commercial radionuclide calibrators is essential when they are used in a clinical setting. Past research [33] tested a number of commercial radionuclide calibrators to verify the accuracy of activity measurements when the instrument provided by the manufacturer is used without change. The study showed that some instruments met the 5% specification on accuracy for a range of radionuclides. However, some instruments provided readings that were up to 90% in error, while others showed systematic discrepancies for all radionuclides tested [33]. Therefore, in order to reduce the standard uncertainty *u*_rel_(*A*) associated with activity measurements, there is a continuing need for calibration and testing of clinical instruments using reference standards traceable to national primary standards.

According to (26), clock offsets *T*_0_ − *T*_cal_ between the calibration time of the radionuclide and the acquisition start time may introduce further uncertainty. This statement is particularly true for short half-life diagnostic radiopharmaceuticals such as ^18^F and ^99m^Tc, where the impact of clock offset on the final relative standard uncertainty may be significant (see also Figure 1). For the above reason, an essential requirement for accurate activity administration and quantitative data analysis is that clocks within a nuclear medicine department, within all instruments and all computers, be synchronized and checked daily (or at least weekly). Current EANM procedure guidelines for tumour PET imaging state that, in the case of [18]F-fluorodeoxyglucose (FDG) PET imaging, clocks should be synchronized with the official local time within 1 min [34].

In addition, if the time of activity calibration is temporally distant from the acquisition start time, (26) reveals that the final uncertainty may significantly increase, especially when system calibration is performed with short half-life radionuclides (see the term involving *u*_rel_(*T*_1/2_) in (26)).

Ultimately, the typical relative standard uncertainty *u*_rel_(*T*_1/2_) associated with the half-life of radionuclides used in both diagnostic and therapy is about (and often below) 0.05% [6]. It is worth noticing that a recent study on this subject [35] found that the spread of experimentally determined half-life values for a particular radionuclide may be larger than expected from the claimed accuracies [35–37]. The foremost cause of the tendency to underestimate the uncertainty of experimentally determined half-life values is due to a number of reasons, among which are possible inaccuracies in data analysis [35–37].

To the best of the authors' knowledge, this paper is the first work analyzing how all meaningful sources of uncertainty propagate through the calculation to the combined uncertainty in the calibration factor. In the present research, a simplified formula for the assessment of the combined uncertainty in the calibration factor was derived. We believe that, at a time of increasing need for accuracy in quantification studies, the proposed approach has the potential to be easily implemented in clinical practice.

## 4. Final Message

As a final message we note that in recent years there has been an increase in the development and use of radiopharmaceuticals for molecular radiotherapy (MRT). Within this context internal dosimetry has become a central issue in MRT, as there is increasing evidence that treatment outcome is related to the absorbed doses delivered to tumours and to normal organs rather than to the administered activities [38]. This statement is particularly relevant in the light of the recent EC Directive 2013/59/EURATOM, Article 56, which states that individual dose planning for radiotherapy patients (including MRT) must be enforced in legislation by EU member states by 6 February 2018. As a consequence, within the next few years, internal dosimetry is set to become an important component in MRT clinical practice.

System calibration is a central issue in MRT as accurate activity quantification is required to achieve reliable dosimetry. The current state of the art for quantitative activity measurement is the use of combined SPECT-CT or PET-CT imaging to provide a 3D distribution of activity within the patient. To give absolute activity, the system must be calibrated and each image must be corrected for a number of degrading effects (e.g., scatter, attenuation, partial volume effect, and dead time). In spite of the increasing awareness that an accurate assessment of the absorbed dose to critical tissues would provide a more effective targeted use of MRT, there are no validated standard protocols or any established methods for calibration or verification of system performance.

Against this backdrop, an IAEA Coordinated Research Project was begun in 2009 (E21007 Development of Quantitative Nuclear Medicine Imaging for Patient Specific Dosimetry) with the purpose of addressing the lack of harmonized protocols or guidelines. Another research project, funded by the European Metrology Research Programme (EMRP) and finished in 2015, aimed to develop and improve the standards and calibration methods for measuring radioactivity, quantitative imaging, and dose calculations [32]. The project, named MetroMRT (Metrology for Molecular Radiotherapy), formulated MRT dosimetry as a measurement chain that is traceable to primary standards. The links in the chain are (I) measurement of administered activity; (II) sequence of activity measurements through quantitative imaging procedures; (III) construction of an activity-time function from the sequence; (IV) integration of the activity-time function; and (V) calculation of the absorbed dose from the activity-time integral.

Of note, one of the main aims of the project was to investigate the uncertainties entailed in each step of the measurement chain. Ultimately, an EMPIR-funded joint research project begun in 2016, named “metrology for clinical implementation of dosimetry in molecular radiotherapy” (MRTDosimetry, [29]), is aiming to provide the metrology for the clinical implementation of absorbed dose calculations in MRT. The project is building on the results and outputs from the preceding EMRP MetroMRT project.

In conclusion, the MRT community has an urgent need for dosimetry calibration standards, validation methods, and clear guidance on how to implement MRT dosimetry in every European clinic offering MRT. Comprehensive guidance has yet to be presented in this field and there is no doubt that internationally endorsed recommendations for good practice would lead to further advances in this area.

## Figures and Tables

**Table 1:** Half-lives and associated standard uncertainties for a selection of radionuclides used both in diagnosis and in therapy [6].

Radionuclide	*T*_1/2_(*u*(*T*_1/2_))	*u*_rel_(*T*_1/2_)/%	Use
^18^F	1.82890(23) h	0.012	Diagnosis
^99m^Tc	6.0067(10) h	0.017	Diagnosis
^131^I	8.0233(19) d	0.023	Diagnosis and therapy
^177^Lu	6.647(4) d	0.060	Therapy
^90^Y	2.6684(13) d	0.048	Therapy
^223^Ra	11.43(3) d	0.26	Therapy
